# Noncoding RNAs implication in cardiovascular diseases in the COVID-19 era

**DOI:** 10.1186/s12967-020-02582-8

**Published:** 2020-10-31

**Authors:** S. Greco, A. Madè, C. Gaetano, Y. Devaux, C. Emanueli, F. Martelli

**Affiliations:** 1 Molecular Cardiology Laboratory, IRCCS Policlinico San Donato, San Donato Milanese, 20097 Milan, Italy; 2 Laboratory of Epigenetics, Istituti Clinici Scientifici Maugeri IRCCS, 27100 Pavia, Italy; 3 Cardiovascular Research Unit, Luxembourg Institute of Health, Strassen, Luxembourg; 4 Imperial College London, National Heart and Lung Institute, Hammersmith Campus, London, W12 0NN UK

**Keywords:** COVID-19,, SARS-CoV-2,, Cardiovascular disease, Noncoding RNAs,, Transcriptomics

## Abstract

COronaVIrus Disease 19 (COVID-19) is caused by the infection of the Severe Acute Respiratory Syndrome CoronaVirus 2 (SARS-CoV-2). Although the main clinical manifestations of COVID-19 are respiratory, many patients also display acute myocardial injury and chronic damage to the cardiovascular system. Understanding both direct and indirect damage caused to the heart and the vascular system by SARS-CoV-2 infection is necessary to identify optimal clinical care strategies. The homeostasis of the cardiovascular system requires a tight regulation of the gene expression, which is controlled by multiple types of RNA molecules, including RNA encoding proteins (messenger RNAs) (mRNAs) and those lacking protein-coding potential, the noncoding-RNAs. In the last few years, dysregulation of noncoding-RNAs has emerged as a crucial component in the pathophysiology of virtually all cardiovascular diseases. Here we will discuss the potential role of noncoding RNAs in COVID-19 disease mechanisms and their possible use as biomarkers of clinical use.

## Introduction

The novel human Severe Acute Respiratory Syndrome CoronaVirus 2 (SARS-CoV-2), isolated on 7th January 2020, has been identified as the cause of acute respiratory distress syndrome (ARDS) cases of unknown etiology detected in Wuhan City, Hubei province of China and then indicated as Coronavirus Disease 2019 (COVID-19) [1]. Because of the rapid global spread of the COVID-19, on 11th March 2020, the Director-General of the World Health Organization (WHO) defined the disease as a pandemic [2] and up to October 27th 2020, there have been more than 43 million confirmed cases and more than 1 million deaths worldwide [3].

The main clinical manifestations of COVID-19 are respiratory. However, many patients also display a severe involvement of the cardiovascular system [4–9]. Thus, it is of paramount importance to understand the direct and indirect damage caused to the cardiovascular system by SARS-CoV-2 infection, as well as the underpinning pathogenetic mechanisms.

Here we will review the importance of transcriptomics techniques in our understanding of human coronavirus disease mechanisms in the cardiovascular system and for the identification of biomarkers of potential clinical use. Specifically, we will focus on noncoding RNAs (ncRNAs), an emerging class of regulatory RNAs [10]. Given their fundamental role in gene expression regulation, we propose ncRNAs as promising candidates for understanding the consequences of SARS-CoV-2 infection on the cardiovascular system.

## Noncoding RNAs

Analysis of the human genome has shown that less than 2% is transcribed into protein-coding RNAs (mRNAs), while almost 60% is transcribed into RNAs lacking protein-coding potential (ncRNAs) [11, 12].

Based on their transcript size, ncRNAs can be classified into two groups using 200 nucleotides as length threshold: small noncoding RNAs (small ncRNAs) and long noncoding RNAs (lncRNAs). The most studied small ncRNAs are microRNAs (miRNAs) and long ncRNAs, which  include also circular RNAs (circRNAs) [11, 13].

MicroRNAs (miRNAs) are short noncoding RNAs (18–25 nucleotides) that modulate gene expression by sequence-specific recognition of their target transcripts. Most miRNA genes are transcribed by RNA polymerase II from intergenic, intronic or polycistronic loci forming an intermediate hairpin of 60–70 nucleotide, named ‘‘precursor miRNA’’ (pre-miRNA) [14]. The pre-miRNA is then transported out of the nucleus and cleaved by the cytoplasmic RNase III Dicer into a short miRNA duplex [15, 16]. One strand of this duplex binds to the Argonaute (AGO) protein forming the RNA-induced silencing complex (RISC), which can recognize the target mRNA by sequence complementarity [17, 18]. This binding leads to the degradation and/or translational inhibition of the target mRNAs. Each miRNA can have hundreds of RNA targets and also a single mRNA may have several miRNA recognition sequences, generating complex regulatory networks [19].

LncRNAs are ncRNAs regulating the transcription and translation of protein‐coding gene expression [20–23]. They have some similarities with coding genes, such as the presence of epigenetic marks, introns and the existence of splice variants, and the transcription driven by promoter elements. Moreover, they can be either polyadenylated or non-polyadenylated [24]. They may originate from either the sense or antisense DNA strand, can overlap coding genes entirely or partly. They can be divided in sense, antisense, intronic, intergenic, and bidirectional lncRNAs, enhancer-associated RNAs (eRNAs), and promoter associated long RNAs (PALRs) [25]. Finally, there are circRNAs, that are circularized RNAs generated by back-splicing events of pre-mRNAs [26].

LncRNAs can function as epigenetic regulators, can regulate the transcription rate by assembling transcriptional activators and repressors [27]. Some nuclear lncRNAs have been implicated in maintaining nuclear structures, including interchromatin granules, nuclear speckles and paraspeckles [22]. LncRNAs can also regulate the gene expression by binding to mRNAs and modulating their translation and/or stability [28, 29]. Despite their generally low-abundance, some lncRNAs can accumulate because they high stability and, by sequestering miRNAs, can function as competing endogenous RNAs (ceRNAs) [30, 31].

## Coronavirus biology

**Fig. 1:**
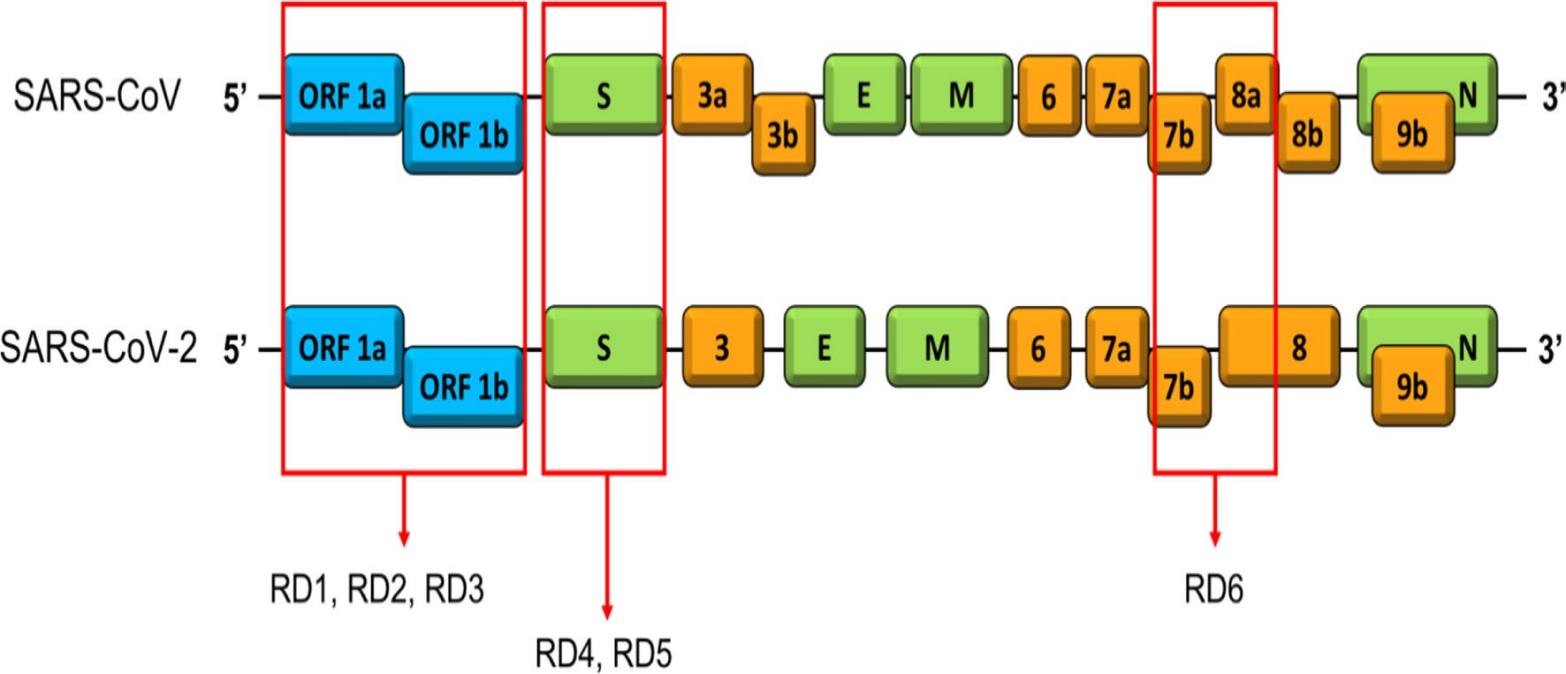
Differences between genome of SARS-CoV and SARS-CoV-2. The six regions of difference (RD) are indicated by red rectangles

Sars-CoV-2 belongs to the Beta-coronavirus genus of the *Coronaviridae* family; like the other members of the family, Sars-CoV-2 is enveloped and its genome is a single-stranded positive-sense RNA of around 30 kb [32]. Coronaviruses genome encodes for nonstructural proteins and for four structural proteins: spike (S), envelope (E), membrane (M) and nucleocapsid (N) proteins (Fig. 1) [33]. The S protein can recognize the receptor of the host cell and is responsible for cell membrane fusion [34], the N protein interacts with the viral RNA to assemble the ribonucleoprotein [35], the E protein is necessary for virion assembly [36] and the M protein has a pivotal role in virus assembly [37].

All the members of the *Coronaviridae* family seem to have the same replication mechanism: the genomic RNA represents the template used to translate two partly overlapping open reading frames in two polyproteins, which encode the nonstructural proteins necessary to the replication-transcription complexes assembly in association with cytoplasmic membranes [32, 38].

Coronaviruses can infect many animal species, causing different symptoms, such as respiratory and intestinal diseases [39, 40]. Among human coronaviruses, severe acute respiratory syndrome-coronavirus (SARS-CoV) and Middle East respiratory syndrome-coronavirus (MERS-CoV) are the best studied, due to the epidemics that originated in 2002 and 2012, respectively [39].

Virology and genetic studies indicate that human coronaviruses have a reservoir in nature not corresponding with the intermediary host species, which is responsible for dissemination to humans [39–41]. Since SARS-CoV-2 genome is more than 96% identical to a bat coronavirus, it is very likely that bats may have been the initial zoonotic host [42]. However, the intermediary species is unknown. The most common human–human transmission is through respiratory droplets generated by sneezing and coughing [43].

Genetic sequence analysis revealed that Sars-CoV-2 has 79.0% nucleotide identity to SARS-CoV and 51.8% identity to MERS-CoV [44, 45]. Comparing Sars-CoV-2 and SARS-CoV genomes, six regions of difference have been recognized (Fig. 1). Based on proteomic comparison, SARS-CoV-2 proteins are highly homologous (about 95%–100%) to the SARS-CoV virus proteins [45]. The similarity of SARS-CoV and SARS-CoV-2 viruses is also confirmed by the fact that they both use angiotensin-converting enzyme 2 (ACE2) as cellular receptor, via the receptor-binding domain of surface spike glycoprotein S [46–48].

For their similar structural and pathogenicity features, the better studied SARS-CoV and MERS-CoV constitute important models when developing hypothesis for SARS-CoV-2 disease mechanisms.

## COVID-19 implication of the cardiovascular system

**Fig. 2:**
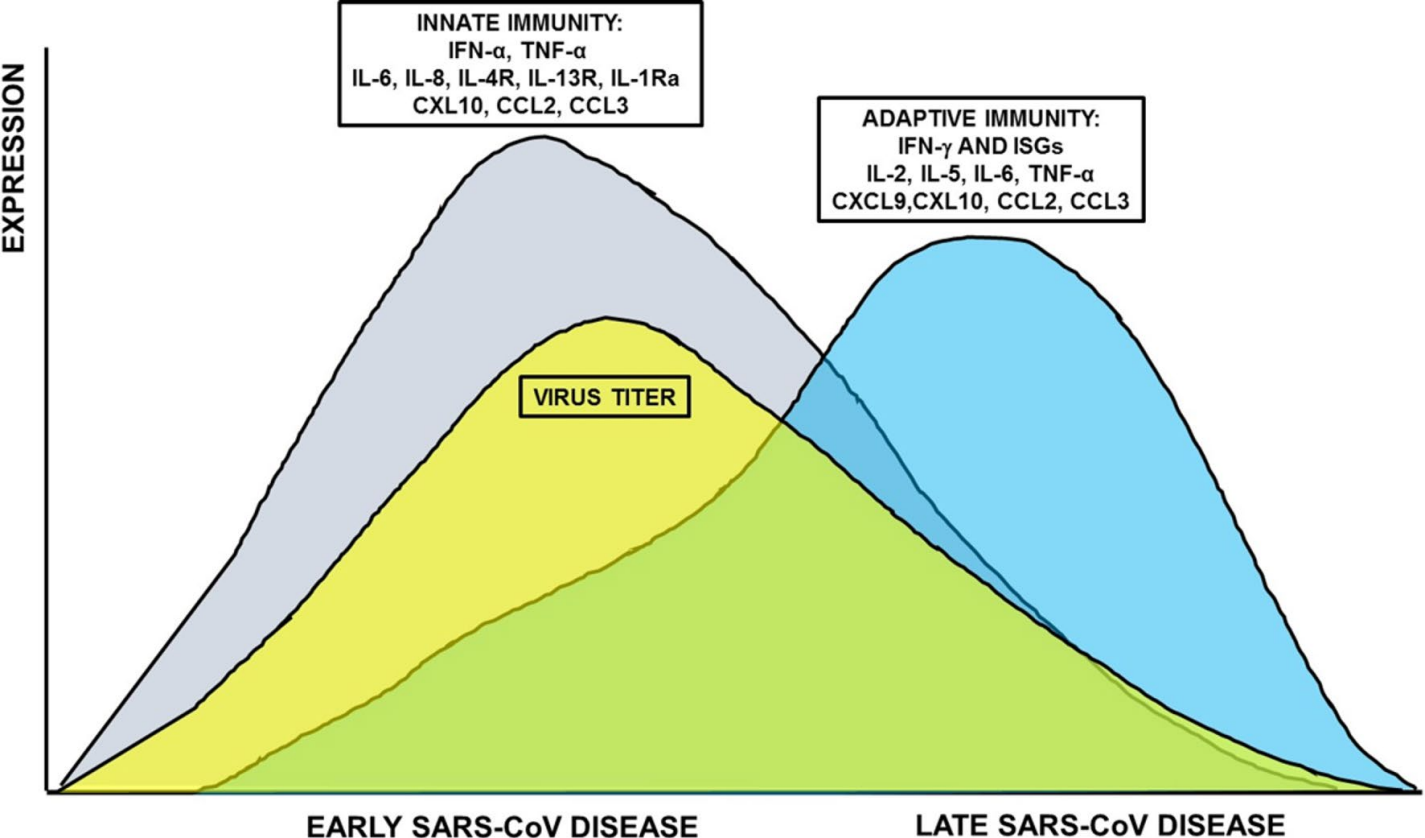
Time course of host responses to SARS-CoV infection in humans. A biphasic expression of inflammatory mediators is associated with the early disease, where the cellular infiltration into the lungs, peaks with the higher viral titer and the late disease that corresponds to viral clearance in non-complicated ARDS. The switch from innate to adaptive immune response is important for fighting ARDS

Patients with pre-existing cardiovascular diseases (CVDs) have a higher risk of severe disease and death upon SARS-CoV-2 infection [4–6, 8]. It is plausible that COVID-19 can affect the cardiovascular system at various levels. Indeed, ACE2 is expressed by a multitude of cell types, including cardiac and vascular cells [49], which may represent direct targets. Accordingly, SARS-CoV-2 infection has been associated with multiple direct and indirect cardiovascular complications including arrhythmias as well as myocardial injury due to hypoxia, microvascular thrombosis and systemic cytokine release syndrome (“cytokine storm”) [8, 50]. Indeed, the immune response is crucial for infection resolution, but this response can also result in immunopathogenesis. During the disease course, SARS-CoV viral loads were observed to decrease while disease severity increased, suggesting that immunopathogenesis may contribute to ARDS [51] and may be an important cause of cardiovascular damage (Fig. 2).

Moreover, antiviral therapies under investigation for COVID-19 may damage the heart and have other cardiovascular side effects, such as arrhythmias and repolarization abnormalities [52].

### SARS-CoV2 and its receptor ACE2

ACE2 is an integral membrane carboxymonopeptidase that has been identified as a functional receptor for coronaviruses, including SARS-CoV and SARS-CoV-2 [48, 53–55]. In this process, it is crucial the S-protein priming by the Transmembrane Serine Protease 2 TMPRSS2 [54].

ACE2 is expressed in alveolar epithelial cells, in line with the respiratory symptoms of COVID-19, but also in other epithelial and non-epithelial cells, in the kidneys and the gut. With relevance to the potential cardiac impact of COVID-19, ACE2 is also expressed by cardiomyocytes, fibroblasts, pericytes, macrophages and the epicardial fat [49, 56–58].

**Fig. 3:**
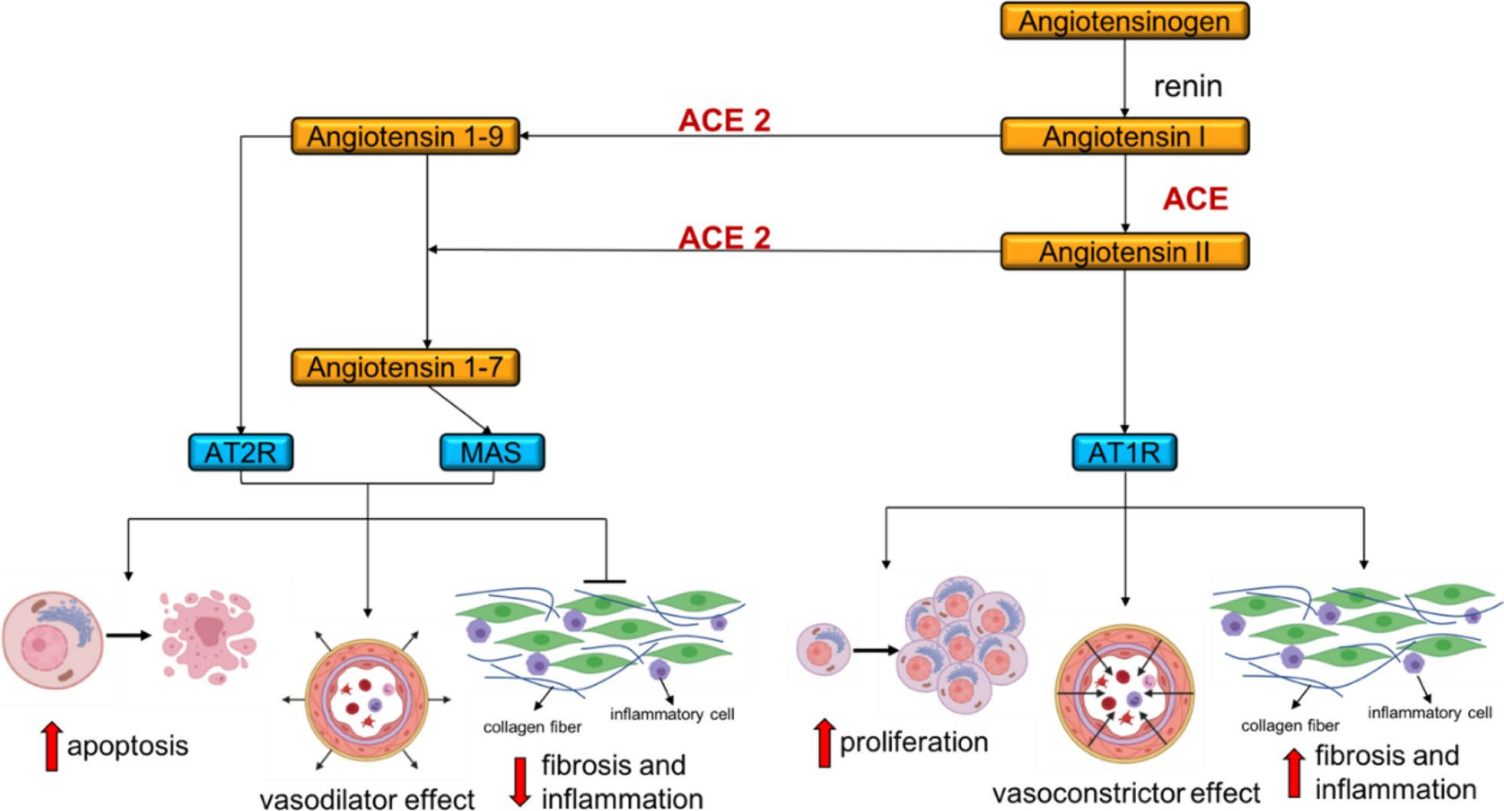
The renin-angiotensin system. ACE2 can cleave both Angiotensin I and Angiotensin II to generate Ang 1-9 and Ang 1-7, respectively. Ang 1-9 and Ang 1-7, in turn, bind Angiotensin II Type 2 Receptor (AT2R) and MAS and have vasodilatory, pro-apoptotic, anti-fibrotic and anti-inflammatory effects. ACE2 converts Angiotensin I to Angiotensin II, which through the binding to AT1R has opposite effects, i.e. vasoconstriction, anti-apoptotic, pro-fibrotic and pro-inflammatory effects

ACE2 is an important member of the renin angiotensin system [54]. As shown in Fig. 2, ACE2 activity is necessary to generate Angiotensin 1-7, which, via the activation of the G protein-coupled MAS receptor, leads to vasodilatory, anti-hypertrophic, and anti-fibrotic effects. This represents an important mechanism to compensate the negative cardiovascular action of an excessive Angiotensin II stimulation on the Angiotensin II Type 1 Receptor (AT1R) (Fig. 3).

In keeping with an important role of ACE2 in the heart, ACE2 knockout mice display impaired cardiac contractility and the upregulation of the hypoxia-associated gene program [58]. In both the mouse model of acute myocardial infarction (AMI) and in idiopathic and ischemic heart failure (HF) patients, increased expression of ACE2 protein and mRNA has been observed [59, 60].

Moreover, ACE2 is a transmembrane protein, whose catalytic domain is located at the extracellular side of the cell and it can be released into the blood after cleavage by ADAM17 (also named TACE). Previous studies have reported increases in circulating ACE2, possibly due to augmented ACE2 shedding, to be associated with cardiovascular risk factors, such as advanced age and diabetes mellitus [61], which are also risk factors for COVID-19 [8, 50].

Given ACE2 expression in the heart, it is conceivable that the SARS-CoVs virus can have a direct effect on cardiac function. Accordingly, in several SARS-CoV patients, viral RNA was detected not only in the lungs, but also in the myocardium [62, 63]. Increased ACE2 expression in cardiovascular patients [59, 60] may increase their susceptibility to SARS-CoVs infection. However, in the heart of both SARS-CoV infected mice and of SARS patients, decreased ACE2-levels were observed [64], suggesting a complex virus/receptor dynamic that needs to be elucidated.

Further complicating the scenario, ACE2 levels can also be increased by inhibitors of the renin angiotensin system [65]. However, while the impact of these drugs on COVID-19 is still largely unknown, a retrospective study did not identify inhibitors of the renin angiotensin system as independent predictors of poor outcome [66, 67].

### Acute cardiac injury

Different studies found that the values of cardiac Troponins were increased in COVID-19 patients with more severe disease [4, 5, 68–70], indicating an association of SARS-CoV-2 with myocardial damage. Indeed, in a study surveying 187 COVID-19 hospitalized patients [9], myocardial injury identified by increased Troponin T and N-terminal Pro Brain Natriuretic Peptide (NT-pro-BNP) levels was significantly associated with death, while the prognosis of patients with underlying CVDs, but without myocardial injury, was less severe. Moreover, cardiac injury was associated with cardiac dysfunction and arrhythmias.

Accordingly, there are several studies reporting a direct effect on heart function of coronaviruses with a pathogenicity similar to that of SARS-CoV-2. In a rabbit model of coronavirus infection, viral-cardiomyopathy and progression into dilated cardiomyopathy (DCM) have been described [71]. Moreover, MERS-CoV infection has been associated with acute myocarditis and HF [72]. Interestingly, in the hearts of both SARS-CoV infected mice and SARS patients, macrophage infiltration with evidence of myocardial damage was observed [64]. A similar pattern with low-grade myocardial inflammation and viral particles in the heart has been reported in one COVID-19 case [73]. Cardiomyocytes showed non-specific features such as focal myofibrillar lysis, and lipid droplets, but no viral particles were observed in myocytes and endothelia, suggesting infected macrophage migration from the lung or during a viraemic phase. Other myocarditis single cases in which endomyocardial biopsy was performed scored negative for SARS-CoV-2 genome presence [74, 75]. Thus, further studies with higher numerosity are definitely needed to ascertain the nature of COVID-19 myocarditis.

Li et al. [76] reported in SARS patients an impairment in left ventricular performance during the acute phase, but reversible on clinical recovery. This impairment was more severe in critically ill patients and elevated lactate dehydrogenase levels, reflecting the severity of tissue damage, were associated with decreased ejection fraction [76].

While the expression of ACE2 is well known in heart, it is controversial the expression of TMPRSS2 [77]. These data draw the attention on the mechanism by which the virus could enter in the cardiomyocytes, and on the existence of secondary effects related to hypoxia and systemic inflammation during complicated COVID-19 courses. Indeed, the finding of a more prominent immune reaction in patients with more critical illness, and the association of cytokines as pro-inflammatory mediators in HF, support the hypothesis that the heart function impairment may be due to the cytokine storm in response to SARS infection [76, 78]. Likewise, also in COVID-19 patients, a cytokine storm triggered by the SARS-CoV2 infection may result in damage to myocardial cells.

This strong inflammatory response may also confer risk for atherosclerotic plaque rupture in coronary artery disease patients, increasing the risk of acute coronary syndrome in more severely affected COVID-19 patients, akin to what has been observed in influenza viral illness [79].

Also important in elevating the risk of cardiac injury is hypoxemia caused by respiratory dysfunction, higher risk of capillary embolism [80] and the increased metabolic demands. Moreover, many antiviral drugs can cause cardiac damage [81], further complicating the clinical situation.

### Cardiac arrhythmia

In hospitalized COVID-19 patients, cardiac arrhythmia was present in more than 15% of the patients and was far more common in those presenting serious symptoms and requiring intensive care [5, 9]. While myocarditis should be considered, high prevalence of arrhythmia might be also attributable to hypoxia, cytokine storm syndrome and metabolic disorder in the setting of viral infection, possibly in the presence of prior CVDs.

Indeed, there are several possible pathophysiological causes of arrhythmias such as: 1) the direct injury to cardiomyocytes that alters the membrane electrical conduction, 2) massive edema, 3) ischemia originating from microvascular disease following the infection, 4) re-entrant arrhythmias caused by myocardial fibrosis or scars and 5) proinflammatory cytokines storm. The first three events could occur in the acute setting, while the fourth and fifth are associated with chronic or healed myocarditis. Moreover, the proinflammatory cytokines storm (e.g., IL-6) might cause alteration in the desmosomal proteins of the cardiomyocyte membrane that could be arrhythmogenic [75, 82].

### Previously existing cardio-metabolic diseases

There is increasing evidence that patients with CVDs and/or diabetes mellitus are more likely to develop severe symptoms when affected by COVID-19 [4–6, 83, 84]. Among COVID-19 patients with severe symptoms, many had hypertension, heart disease and arrhythmia. Acute coronary syndrome seems to have a particularly poor prognosis in COVID-19 patients. Indeed, upon SARS-CoV-2 infection, cardiac insufficiency is more likely to occur in these patients where cardiac function is already compromised by myocardial ischemia [4–6, 83, 84].

Moreover, SARS-CoV infection leads to long term disorders of lipid and glucose metabolism [85]. Considering the similarities between SARS-CoV and SARS-CoV-2, this novel virus might also inflict a similar chronic damage.

## Noncoding RNAs in cardiovascular diseases

Transcriptomics has been extensively used to understand CVD pathogenesis, to identify coding and ncRNAs with a potential as therapeutic targets and clinically useful biomarkers. These studies have been extensively reviewed elsewhere [10, 86–89]. Here we will only give few examples that may be paradigmatic also in the investigation of the cardiovascular implications of COVID-19.

Transcriptomics first focused on mRNAs expressed in the heart, identifying transcripts encoding mostly contractile sarcomeric proteins, cytoskeletal proteins, ion channels, intracellular signal transducers, including apoptosis and cell proliferation genes, and proteins maintaining the redox state of the myocardium [90, 91].

Transcriptomic profiling identified several miRNAs deregulated in left ventricles (LV) of both dilated (DCM) and ischemic cardiomyopathy (ICM) patients [92]. Additional studies allowed identification also of signatures specific to particular cardiomyopathies, such as DCM in patients with reduced catecholamine sensitivity [93], DCM in patients with a reverse-remodeling response following β-blocker treatment [94] and ICM in patients affected by type 2 diabetes mellitus [95].

MiRNA expression is also dysregulated in viral myocarditis [96–98]. In particular, miR-155-5p is one of the miRNAs consistently modulated in both human and mouse viral myocarditis, contributing to myocardial damage through the modulation of monocyte-macrophages cardiac infiltration and T lymphocyte activation [98].

Genome-wide analyses of LV samples of HF patients identified a subset of lncRNAs significantly deregulated compared with healthy controls; despite the limited evolutionary conservation of lncRNAs, many of these findings were also confirmed in relevant mouse models of disease [86, 99–103].

Particularly relevant are circRNAs originating from the multi-exon gene Titin, that are dysregulated in both dilated and hypertrophic cardiomyopathies, and are regulated by the splicing factor RNA binding motif protein 20 (RBM20) [104].

Of great translational relevance is the fact that ncRNAs are released into the blood where they are sufficiently stable to be readily measured with common laboratory techniques, such as qPCR, and that their concentration levels can differentiate diseased patients from healthy subjects. In addition, their measurement requires a minimally invasive procedure, thus representing an enormous reservoir for biomarkers discovery, for both diagnostic and prognostic applications. Many genome-wide profiling studies of circulating ncRNAs have been performed. Among miRNAs, heart and muscle-enriched miRNAs, also named myomiRs (miR-1-3p, miR-133a-3p, miR-133b-3p, miR-208a/b-3p, and miR-499-5p) seem to be particularly relevant [105–110].

Indeed, in patients with AMI, myomiRs have been reported to be elevated in plasma, likely due to cardiomyocyte necrosis and thus released into the circulating system [111, 112]. Accordingly, miR-208a-3p and miR-499-5p were also elevated in the plasma of viral-cardiomyopathy patients and their levels correlated positively with myocardial damage assessed by measuring Troponin T levels [113]. Among myomiRs, miR-208 is of particular interest, since its expression is highly cardiac-specific. Moreover, Voellenkle et al., analyzing the pattern of expression of peripheral blood mononuclear cells (PBMCs) identified a miRNA signature characterizing DCM patients [109].

Also lncRNAs can be found in the peripheral blood and hold a promising biomarker potential. The expression of LIPCAR (long intergenic noncoding RNA predicting cardiac remodeling) in plasma has been found to be correlated to cardiac remodeling progression after AMI [114, 115]. Other potential biomarkers are MIAT (myocardial infarction associated transcript), discriminating ST-elevation from non ST-elevation AMI [116], SENCR (smooth muscle and endothelial cell-enriched migration/differentiation-associated long noncoding RNA), associated to LV remodeling [117], as well as NRON (noncoding repressor of NFAT) and MHRT (Myosin Heavy Chain Associated RNA Transcripts), lncRNAs elevated in HF patients and independent predictors of HF events [117, 118]. Greco et al. [102], analyzing ICM patients found that ANRIL, HOTAIR, and LOC285194/TUSC7 had similar modulation in PBMC and heart tissue, suggesting a potential role as functional biomarkers.

**Fig. 4:**
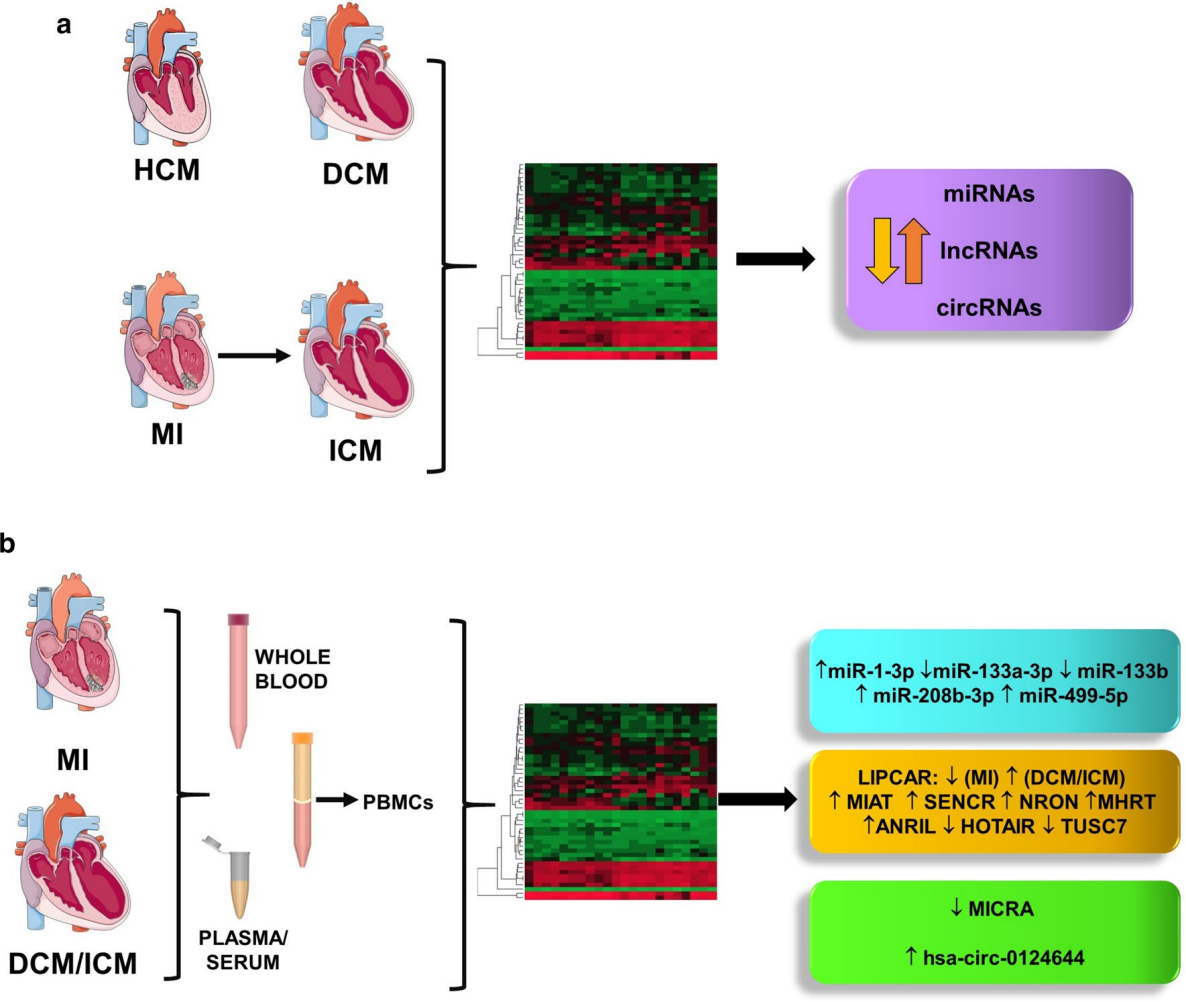
Noncoding RNAs dysregulation studied by genome-wide transcriptomic analysis. **a** Analysis of the molecular modifications underlying both cardiac hypertropy (HCM) and remodeling (DCM and ICM) allowed the identification of dysregulated miRNAs, lncRNAs and circRNAs in the heart. **b** The release of ncRNAs by diseased cardiac tissues in the peripheral blood and the immunomodulation associated to CVD allowed the identification of dysregulated ncRNAs in whole blood, plasma/serum or in PBMCs, to be used as potential biomarkers

Among circRNAs, MICRA (myocardial infarction-associated circular RNA) levels in the blood are associated with ischemic HF [119], while hsa_circ_0124644 and hsa-circ-0005870 are potential diagnostic biomarkers of coronary artery disease [120] and hypertension [121] respectively (Fig. 4b).

## Transcriptomics studies of SARS-CoVs infection: focus on inflammation

The SARS-CoV-2 host-response transcriptomics changes have just started to be investigated, but reports on them are increasing over time.

### Peripheral blood

The gene expression profile of blood samples derived from COVID-19 patients not only allows the definition of the host responses to the infection and, thus, a better understanding of the disease pathogenesis, but also the identification of potential biomarkers that could help monitoring patient responses to the disease.

Specifically, a distinct pattern of inflammatory cytokines was identified in bronchoalveolar lavage fluid and in the peripheral blood mononuclear cells, including CCL-2, -3 and -4, and CXCL-10, as well as the activation of the P53 signaling-pathway in lymphocytes that may be related to COVID-19 lymphopenia [122]. In addition, several differential expressed transcripts were identified in PBMCs of COVID-19 patients with severe or mild symptoms [123] compared to controls. Cytokine-mediated signaling, the natural killer cell mediated toxicity and T cell activation pathways were enriched terms in common between the two disease stages, while Interleukins- and TNF-signaling pathways were enriched terms specific for COVID-19 patients with severe symptoms [123].

Moreover, the single-cell RNA-sequencing (scRNA-seq) approach has been used to profile the SARS-CoV-2 host-response in the PBMCs of COVID-19 patients, and to comprehensively characterize the immunological changes [124–130].

In eight COVID-19 patients, scRNA-seq of PBMC indicated the depletion of the innate immune subsets compared to healthy PBMC [125]. In addition, a new population, which was annotated as ‘developing neutrophils’, has been found increased only in patients with ARDS; this population expressed several genes in common with the neutrophil progenitors, but not canonical neutrophil markers, indicating that they could be neutrophils at various developmental stages [125]. The monocyte subset was enriched in CD14, but without a substantial expression of pro-inflammatory cytokine genes (TNF, IL6, IL1B, CCL3, CCL4 or CXCL2), which is in contrast with other reports [124, 126]. Additionally, CD14 + monocytes showed the upregulation of a signature of interferon (IFN)-stimulated genes that correlated with clinically relevant parameters [125]. This strong activation of interferon-stimulated genes was also confirmed by Arunachalam et al. [127], primarily in patients with mild or moderate disease, that is associated to reduced expression of genes encoding proinflammatory cytokines [127]. Similarly, a more pronounced expression of type I IFN in mild COVID-19 patients and lack of IL1B mRNA levels increase were also confirmed in a recent study that analyzed by scRNA-seq two independent large cohorts of COVID-19 patients [128].

Conversely, an increased TNF/IL-1β-driven inflammatory response was observed in severe COVID-19 patients as compared to severe influenza, and to mild COVID-19 patients [129].

The scRNA-seq approach has also been used to study the immune cell landscape of two severe-stage COVID-19 patients prior to and following tocilizumab-induced remission [126]. In this study, a monocyte subpopulation that contributes to the inflammatory cytokine storms was identified. Bioinformatics analysis predicted a severe stage-specific interaction-networks formed by monocyte receptors and cytokines [126]. In addition, tocilizumab treatment in severe-stage COVID-19 patients led to an increased number of antibody-secreting plasma B cells, while CD8 + T cell-related cytotoxicity and cytokine production did not change [126]. These data provide insights into the understanding of the inflammatory storms observed in COVID-19 patients and identify also potential drug targets.

Zhang et al. analyzed by scRNA-seq the PBMCs of thirteen COVID-19 patients with clinical conditions ranging from moderate to severe and to convalescent [130]. Differentially expressed genes of CD14 + and CD16 + COVID-19 monocytes, especially in severe COVID-19 state, were associated with IFN responses, myeloid leukocyte activation, cytokine production and nuclear factor (NF)-κB signaling pathway [130]. Interestingly, in patients at the early phase of convalescence, despite of the recovery of most of the clinical parameters to a normal range, the immune system was still fully activated, as demonstrated by a still high naive T and T_reg_ subsets ratios [130].

Early and late stages of recovery have been analyzed in detail by Wen et al., confirming that COVID-19 patients are still vulnerable after hospital discharge [124]. COVID-19 early recovery stage was characterized by CD14 ++ monocytes with high inflammatory gene expression as well as by the abundance of CD14 ++IL1β + cells, while T cells decreased remarkably, compared with both late recovery stage and healthy control subjects.

### Tissues

Due to the limited cardiac tissue availability, data on SARS-CoV-2-mediated heart transcriptome changes have not been reported yet. However, SARS-CoV-2 infection of human induced pluripotent stem cell-derived cardiomyocytes (iPSC-CMs) induced cytotoxic effects and RNA-seq findings highlighted significant transcriptional changes in gene pathways related to cellular metabolism and immune response [131–133].

Indeed, Sharma et al. [131] observed in infected iPSC-CMs a downregulation of transcriptional pathways related to mitochondrial function, oxidative phosphorylation, and cardiac function, whereas upregulated pathways included immune cytokines, immunomodulators, antiviral response, and apoptosis [131].

Accordingly, Bojkova and collaborators showed that SARS-COV-2 infection of iPSC-CMs induced cytotoxic and proapoptotic effects and abolished cardiomyocyte beating [132]. Virus infection produced a transcriptional response including the up-regulation of genes associated to viral response and interferon signaling, apoptosis and reactive oxygen stress [132]. On the same line, Pérez-Bermejo et al. [133], found that human iPSC-CMs exposed to SARS-CoV-2 demonstrated a productive infection and morphological signatures of damage, which included a distinct pattern of myofibrillar fragmentation and numerous iPSC-CMs lacking nuclear DNA. These morphological changes were also confirmed in human autopsy specimens from COVID-19 patients [133]. RNA-seq transcriptomic data obtained in infected iPSC-CMs suggested a compensatory overexpression of myosin heavy chain genes in response to targeted degradation and also a significant depression of the ubiquitin–proteasome system upon infection [133].

As for the heart, a limited number of reports on lung transcriptomic changes mediated by SARS-CoV-2 infections has been published so far. One of the first studies on lung cells was the analysis of the transcriptional footprint in post-mortem lung samples of COVID-19 patients [134]. In this study a reduced IFN-I and -III response and a consistent chemokine signature compared to other respiratory viruses were observed [134].

The reanalysis of a previously reported dataset identified, as expected, upregulated expression of chemokines and neutrophils in the lung tissue and bronchoalveolar lavage fluid of COVID-19 patients and, in addition, the upregulation of genes coordinating heme biosynthesis [135]. This effect, which has been shown in sepsis secondary to pneumonia to have a protective role against oxidative stress [136], could be responsible of pro-inflammatory cytokine production amplification [137] or cause intravascular coagulation [138].

The possible involvement of altered coagulation following SARS-CoV-2 infection has been also proposed by a study analyzing three publicly available RNA sequencing datasets obtained from clinically isolated samples of bronchoalveolar lavage fluid, PBMCs and from in vitro SARS-CoV-2 infected primary normal human bronchial epithelial cells compared to the respective controls [139]. Gene expression analysis of both bronchoalveolar lavage and bronchial epithelial cells, but not of PBMCs, highlighted the activation of the extrinsic blood coagulation cascade and the suppression of the plasminogen activation system [139].

Moreover, a study performed in autopsy lung specimens from patients who succumbed to SARS-CoV-2 infection supported two phases of disease evolution in patients with severe COVID-19 pneumonia [140]. In the first phase, high expression of IFN pathway genes and of endothelial genes related to tissue damage and fibrosis were observed [140]. This first phase was morphologically characterized by high viral RNA, a histological pattern of exudative diffuse alveolar damage, a shorter disease duration, while the second phase showed a low (or undetectable) viral RNA, and an organizing form of diffuse alveolar damage [140].

Of note, the aforementioned pro-inflammatory cytokines, such as TNF-α, IFN-γ, IL-1β, IL-6, IL-17, and IL-18, are also elevated in HF and in viral myocarditis, and their sustained elevation correlates to HF progression. They are responsible for both compensatory cardiac hypertrophy and fibrosis in the setting of cardiac injury and induce further inflammation [141]. There are contrasting effects attributed to IFN-γ in the heart. Upon adverse stimuli, such as myocarditis or hypertension, the release of IFN-γ by recruited inflammatory cells to the heart results in cardiac fibrosis and hypertrophy. However, other studies have found that IFN-γ has also protective effects, limiting cardiac hypertrophy [142]. The signaling of the pro-inflammatory cytokines is counterbalanced by the release of anti-inflammatory cytokines and TGF-β, which mitigate hypertrophic cardiac remodeling [141].

These data indicate that, along with the direct effect mediated by the virus, such as in myocarditis, the cardiac sequelae may be due to the cytokines storm following the infection, which reinforces the cytokines release, observed in dilated cardiomyopathies.

## Noncoding RNAs regulating inflammation and the cardiovascular system: are they playing a role in COVID-19?

An over-activation of the inflammatory response and the ensuing cytokine storm seem to be a crucial pathogenetic mechanism in COVID-19 patients and, as illustrated in Sect. “Transcriptomics studies of SARS-CoVs infection: focus on inflammation”, the innate immune response pathway has emerged as profoundly dysregulated in SARS-CoVs infections.

The immune system contributes to heart development, composition and function and, in specific circumstances, immune cells can also cause damage, participating to cardiac disease [143]. For instance, in patients with HF, a chronic activation of the innate immune system is often observed, with T-cells and macrophages myocardial infiltration and increased pro-inflammatory cytokine levels (i.e. TNF-a, IL-1b, and IL-6) [144].

NcRNAs are important regulators of these processes and we propose that ncRNAs may play a fundamental role also in the cardiovascular dysfunctions of COVID-19 patients.

As mentioned above, the expression of ACE2 is well known in adult cardiomyocytes [48, 53–55, 77], as well as in neonatal rat cardiomyocytes and in human IPSC-CMs [145]. Bioinformatics analysis predicted miR-200b, miR-200c and miR-429 among the miRNAs targeting ACE2, and in vitro experiments demonstrated that miR-200c modulation regulated the expression of ACE2 [145]. MiR-200c is upregulated by oxidative stress [146] and is also involved in CVDs [146], suggesting that miR-200c-mediated regulation of ACE2 may be important for SARS-CoV-2 entry.

Computational analyses have predicted that SARS-CoV-2 can divert the cellular miRNAs from their transcriptional regulating activity, contributing to the abnormal immunity activation in patients with COVID-19 [147], or synthesizes its own viral miRNAs to reduce the host cell apoptosis preventing host defense [147–149].

On the other side, host miRNAs were predicted to target SARS-CoV-2 spike proteins, suggesting a potential role as therapeutic molecules [149, 150].

**Fig. 5:**
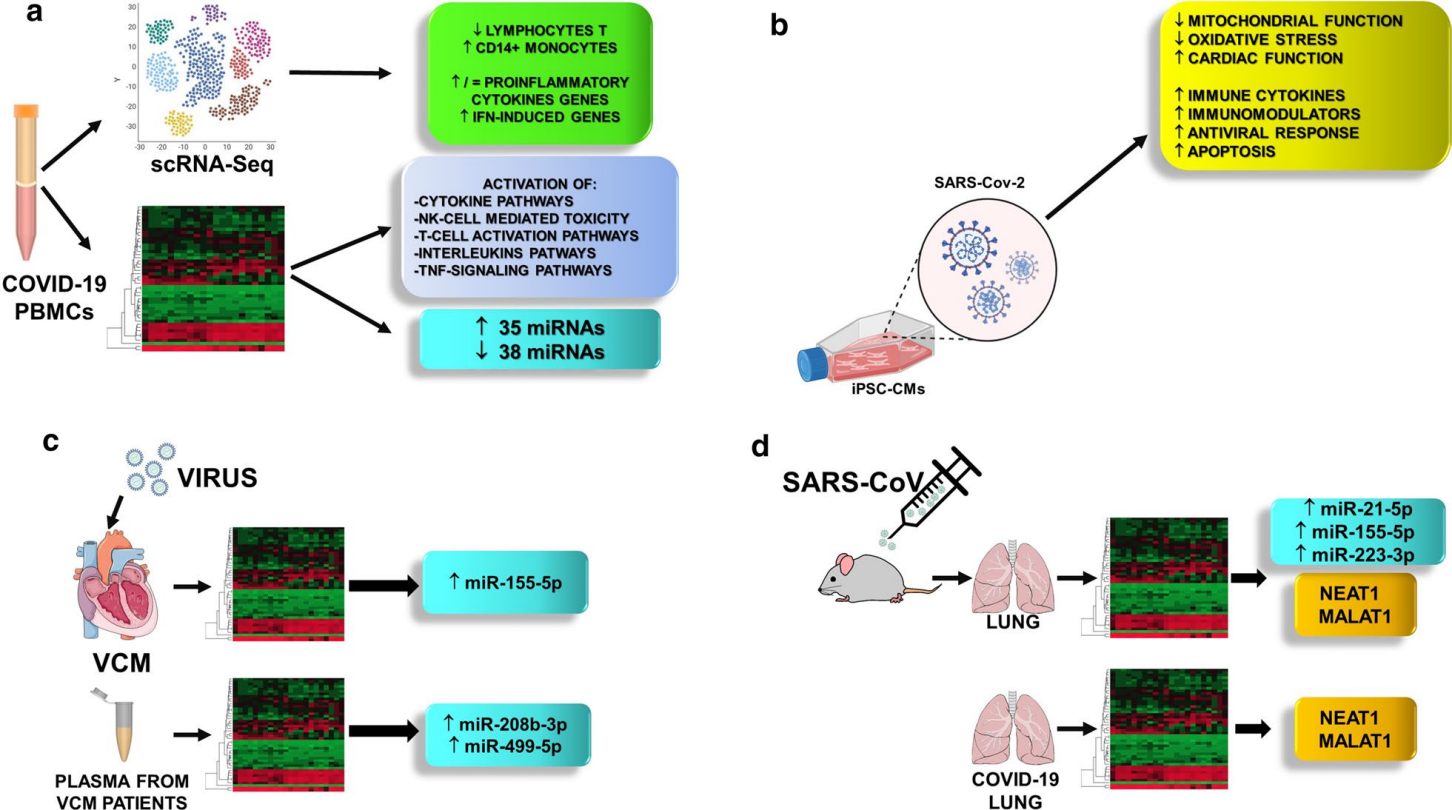
High-throughput analysis of transcriptomic changes induced by viral infection. Transcriptomic changes analyzed by single-cell RNA-seq analysis and bulk RNA-Seq. in **a** PBMCs from COVID-19 patients; **b** iPSC-derived cardiomyocytes infected in vitro by SARS-CoV2 virus. **c** Transcriptomic analysis of endomyocardial biopsies and plasma of viral-cardiomyopathy patients identified miRNAs with an established role also in CVDs. The main miRNAs and lncRNAs identified are shown. **d** Transcriptomic changes in the lungs of SARS-CoV infected mice or in lung biopsies from COVID-19 patients. Specific miRNAs and lncRNAs were shown to be differential expressed

Direct or indirect effects of SARS-CoV-2 infection can alter host response and produce noncoding RNA differential expression. In particular, in bronchial epithelial cells infected with SARS-Cov-2, a complex bioinformatics and computational pipeline, revealed several activated networks, including those involved in immunoglobulin G and interferon lambda [151]. In addition, acute inflammatory response and activation of TNF were also observed. This analysis also revealed several host-derived lncRNAs differentially expressed in COVID-19 patient-derived lung tissue, and in SARS-CoV-2 infected epithelial cells, including MALAT1 (metastasis-associated lung adenocarcinoma transcript 1) and NEAT1 (nuclear-enriched autosomal transcript 1) [151] (Fig. 5).

The computational reanalysis of RNA-Seq dataset of SARS-CoV-2 infected bronchial epithelial cells [134] identified several protein-coding RNAs and lncRNAs differentially modulated [152]. The interaction of lncRNAs with the differentially expressed protein-coding genes was analyzed by network enrichment analysis indicating significant interactions involved in cellular signaling, metabolism, immune response and RNA homeostasis [152].

The RNA-Seq of RNA extracted from peripheral blood samples from 10 COVID-19 patients compared to 4 controls demonstrated that 35 miRNAs were upregulated and 38 miRNAs were downregulated in the human patients with COVID-19 [153]. Enrichment analysis of differentially expressed miRNA target genes revealed that peptidases, protein kinases, and the ubiquitin system were shown to be the highest enrichment categories [153] (Fig. 5).

There are also some hints provided by transcriptomics studies on SARS-CoV. When this infection had a multi-country outbreak in 2002 to 2003, coding RNAs were still the focus of attention for the scientific community and microarrays were the preferential profiling tool. Nevertheless, some studies performed in the following years on lung samples or on bronchoalveolar stem cells collected from mouse infected with adapted SARS-CoV are available [154–157]. These studies identified lncRNAs and miRNAs involved in innate immune response in CVDs. In particular, the lncRNAs Neat1 (nuclear paraspeckle assembly transcript 1) and Malat1 (metastasis-associated lung adenocarcinoma transcript 1) [154] were up- and down-regulated after infection, respectively, and miR-155-5p [156], miR-21-5p and miR-223-3p were among the most deregulated miRNAs upon SARS-CoV infection (Fig. 5).

MiR-155-5p, miR-223-3p and miR-21-5p are expressed in immune cells and involved in both innate immune response [158, 159] and in myocardial infarction or HF [10, 86–88, 158–161]. These miRNAs control the production and secretion of pro-inflammatory cytokines in the heart by Toll-like (TRL) receptors and their downstream signaling pathway, which involves the transcription nuclear factor-kappa B (NF-κB). Indeed, miR-155-5p, along with miR-146a-5p, represents a unique regulatory network for the fine-tuning of the macrophage inflammatory response via regulation of NF-κB activity [162]. In particular, the activation of NF-κB by a stressor stimulates miR-155-5p expression which, amplifying NF-κB activity, enables a strong macrophage activation. As the inflammatory response develops, miR-146a-5p transcription increases, inhibiting its targets IRAK1 and TRAF6, whereby dampening NF-κB activation (Fig. 5).

In addition, also miR-21-5p targets a component of the NF-κB pathway, PDCD4 (Programmed Cell Death 4), thus stimulating the release of pro-inflammatory cytokines and inhibiting the release of anti-inflammatory cytokines [163, 164].

Finally, miR-223-3p seems to have cardio-protective and anti-inflammatory roles by enhancing glucose metabolism and inhibiting granulocyte activation. Accordingly, a decrease of miR-223-3p levels in tissue and plasma has been observed in diabetes and CVDs [95, 165–167].

Another ncRNA of relevance in cardio-immunology is the lncRNA NEAT1, which was reported to induce intima thickening in vascular smooth muscle cells [168]. NEAT1 is also part of the innate immune response by promoting the activation of the inflammasome in macrophages and the release of IL-1β [169].

Together, these data indicate that some of the ncRNAs reported to be dysregulated by transcriptomic profiling in CVDs, are also involved in viral innate immune responses, and that they may be identified as candidate transcripts for our query in understanding the pathogenesis of COVID-19.

## Conclusions and perspectives

Investigating the interplay between SARS-CoV-2, the host antiviral defenses and the cardiovascular system, is fundamental to understand the viral pathogenesis and the infection outcome. Indications from SARS-CoV and other coronaviruses are very helpful; however, SARS-CoV-2 is a novel human pathogen and many aspects of its interaction with the host could be unique.

It is now clear that pre-existing CVDs increase both the severity of the primary respiratory syndrome and the risk of adverse outcomes. SARS-CoV-2 infection consequences on the cardiovascular system should be investigated both for their acute and prolonged sequelae. In this view, transcriptomics may be a powerful approach to study the ncRNA involvement in the disease mechanisms and for the identification of biomarkers.

Significant hurdles are represented by difficulties in measuring and studying ncRNAs due to low abundance of many of them or to specific structural features (e.g. circRNAs). Identification of RNA-based biomarkers and targets requires heavy reliance on relatively expensive sequencing approaches that still lack universally adopted standard operative procedures [10, 23, 170]. Insufficient structural and functional annotation of the noncoding genome is also a significant problem. These limitations should be overcome in order to make significant scientific progresses in our understanding of COVID-19 pathogenesis, facilitating prognosis and hopefully, paving the way to potential therapeutic approaches.

In this respect, international cooperation to share knowledge, patient samples and data collection across many institutes and countries seems as an almost obligatory strategy [171].

## Data Availability

Not applicable.

## Abbreviations

ACE2: Angiotensin-converting enzyme 2
AMI: Acute myocardial infarction
ARDS: Acute respiratory distress syndrome
circRNAs: Circular RNAs
COVID-19: CoronaVIrus Disease 19
CVDs: Cardiovascular diseases
DCM: Dilated cardiomyopathy
HF: Heart failure
ICM: Ischemic cardiomyopathy
IFN: Interferon
IL: Interleukin
lncRNAs: Long noncoding RNAs
MERS-CoV: Middle East respiratory syndrome-coronavirus
miRNAs: MicroRNAs
mRNAs: Messenger RNAs
ncRNAs: Noncoding RNAs
NF-kb: Nuclear factor-kappa B
PBMCs: Peripheral blood mononuclear cells
SARS-CoV: Severe acute respiratory syndrome-coronavirus
STAT1: Signal transducer and activator of transcription 1
TGF: Transforming growth factor
TNF-α: Tumor necrosis factor-α
